# Metabolically similar cohorts of bacteria exhibit strong cooccurrence patterns with diet items and eukaryotic microbes in lizard guts

**DOI:** 10.1002/ece3.5691

**Published:** 2019-10-23

**Authors:** Iris A. Holmes, Ivan V. Monagan, Daniel L. Rabosky, Alison R. Davis Rabosky

**Affiliations:** ^1^ Museum of Zoology & Department of Ecology and Evolutionary Biology University of Michigan Ann Arbor MI USA; ^2^ Division of Herpetology American Museum of Natural History New York NY USA; ^3^ Department of Ecology, Evolution, and Environmental Biology Columbia University New York NY USA

**Keywords:** diet, fungi, gut microbiome, lizard, metabolism

## Abstract

Gut microbiomes perform essential services for their hosts, including helping them to digest food and manage pathogens and parasites. Performing these services requires a diverse and constantly changing set of metabolic functions from the bacteria in the microbiome. The metabolic repertoire of the microbiome is ultimately dependent on the outcomes of the ecological interactions of its member microbes, as these interactions in part determine the taxonomic composition of the microbiome. The ecological processes that underpin the microbiome's ability to handle a variety of metabolic challenges might involve rapid turnover of the gut microbiome in response to new metabolic challenges, or it might entail maintaining sufficient diversity in the microbiome that any new metabolic demands can be met from an existing set of bacteria. To differentiate between these scenarios, we examine the gut bacteria and resident eukaryotes of two generalist‐insectivore lizards, while simultaneously identifying the arthropod prey each lizard was digesting at the time of sampling. We find that the cohorts of bacteria that occur significantly more or less often than expected with arthropod diet items or eukaryotes include bacterial species that are highly similar to each other metabolically. This pattern in the bacterial microbiome could represent an early step in the taxonomic shifts in bacterial microbiome that occur when host lineages change their diet niche over evolutionary timescales.

## Graphical abstract

The ecological processes that underpin the gut microbiome's metabolic response might involve rapid turnover of the gut microbiome in response to new metabolic challenges, or it might entail maintaining sufficient diversity in the microbiome that any new metabolic demands can be met from an existing set of bacteria. We find that the cohorts of bacteria that occur significantly more or less often than expected with arthropod diet items or eukaryotes in lizard hosts include fewer bacterial species than would be expected by chance, but that those species are highly similar to each other metabolically.

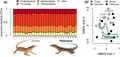

## 1 INTRODUCTION

The ecological community of bacteria that composes vertebrate gut microbiomes performs a set of metabolic functions (Coyte, Schluter, & Foster, 2015; Douglas & Werren, 2016), some of which benefit their host (Foster, Schluter, Coyte, & Rakoff‐Nahoum, 2017; Hanning & Diaz‐Sanchez, 2015). Since gut microbiomes aid in digestion (Rowland et al., 2018) and pathogen resistance (Stough et al., 2016), they are an essential mediators of their host's interaction with its broader biotic community. As such, characterizing the within‐microbiome ecological interactions that allow gut microbes to provide metabolic services to their host is the key to forming a predictive framework for how vertebrates interact with their environment (Alberdi, Aizpurua, Bohmann, Zepeda‐Mendoza, & Gilbert, 2016).

Through their interaction with their hosts, microbiome function can affect entire ecological communities. Epidemic disease in a single species can have ecosystem‐scale effects (McKenna et al., 2010; Olofsson et al., 2016); since microbiomes form part of a host's response to parasites and pathogens, they have the potential to alter the course of epidemic disease outbreaks. Gut microbiomes can also help their host adapt to new food sources (Amato et al., 2015; Delsuc et al., 2014; Hammer & Bowers, 2015), thereby affecting predator–prey interactions, which in turn can have community‐wide effects (Barrios‐O'Neill, Bertolini, & Collins, 2017).

In addition to their importance to macroscale biotic interactions, microbiomes are self‐contained ecological communities (Näpflin & Schmid‐Hempel, 2018). Gut microbiomes are ideal model systems for community ecology and ecoevolutionary interactions because energy inputs come only through the host diet (Amato et al., 2015; Hammer & Bowers, 2015), and because work on bacterial metabolism makes identifying community function straightforward (Franzosa et al., 2014) relative to the empirical and philosophical challenges of quantifying functional profiles in macroscale communities (Funk et al., 2017).

Here, we test hypotheses about the ecological interactions that underpin functional profile in the gut microbiomes of two lizard host species. Both species are generalist insectivores that reproduce oviparously. These traits should lead to similarities in how both host species recruit and maintain their gut microbiome bacteria (Colston, 2017; Kohl et al., 2017). However, they are distantly related phylogenetically, which is likely to lead to differences in their gut microbiome composition (Ley et al., 2008). Given the phylogenetic distance and ecological similarity in our study species, similar responses to diet items and eukaryotes in both species should provide strong evidence for convergent microbial function.

Squamate reptile gut microbiomes are a relatively understudied group compared to other vertebrates (Colston & Jackson, 2016). Available studies indicate that squamate microbiome communities are more similar to those of fish and birds than they are to those of mammals (Colston & Jackson, 2016). Wild squamate gut microbiomes are dominated by bacteria from the phylum Proteobacteria, particularly in the small intestine and cloaca, while the large intestine is more phylogenetically diverse (Colston, Noonan, & Jackson, 2015). Squamate reptiles likely assemble their microbiome through horizontal transmission during interaction with other organisms, including predatory encounters, and from their environment (Colston, 2017; Kohl et al., 2017).

In this paper, we use DNA metabarcoding to identify bacteria, eukaryotes, and diet items in the host's hindguts (Dollive et al., 2013; Kartzinel & Pringle, 2015; Kozich, Westcott, Baxter, Highlander, & Schloss, 2013). While we are agnostic about the health effects of the eukaryotes we detect, they include taxa that are known to cause negative health and fitness effects for their hosts (Li et al., 2018), and so may serve as a proxy for parasitic species. Using community ecology analyses, we identify ecological interactions between all taxa, and test whether bacteria that interact with a diet item or eukaryote have a consistent metabolic profile (Kanehisa, Sato, Kawashima, Furumichi, & Tanabe, 2016). We test three hypotheses about the ecological interactions that underpin metabolic service delivery in response to new diet items or the presence of a eukaryote.

1. The microbiome might turn over completely with each new diet item or eukaryote, thereby drastically changing its taxonomic and functional profiles.
2. The microbiome might not respond in any measurable way. In this case, we might expect to detect the signature of ecological interactions through random chance, but these “interactions” should not be consistent or predictable.
3. A subset of the bacteria (OTUs) in the microbiome could react to the diet item or eukaryote in a consistent and predictable manner.

From this background, we predict how the host‐microbiome system evolves in response to both ongoing and novel interactions with the host's wider biotic community.

## 2 METHODS

### 2.1 Field collection

We collected scats from two species of insectivorous lizards, the silky anole, *Anolis sericeus,* and the rainbow ameiva, *Holcosus undulatus*, from Chiapas, Mexico. Our study site was a coffee farm near remnant evergreen forest patches in a mountainous landscape located at 1,100 m a.s.l. Collection occurred in June and July of 2015. Lizards were captured and held in plastic containers with paper towel substrate for no more than 24 hr. We collected scats from the substrate as they became available and preserved them in 95% ethanol in the field. We held the scats at room temperature in the field and at −20°C once they had been returned to the lab. Further details of capture, study location, and captive husbandry are given in Monagan, Morris, Davis Rabosky, Perfecto, and Vandermeer (2017).

### 2.2 Sample preparation

We extracted total DNA from scat samples using QIAGEN DNEasy spin column kits, with a 12‐hr proteinase‐K digestion. We used a high‐fidelity polymerase to amplify a 16S rDNA V4 amplicon in the bacterial genome, F: GTGCCAGCMGCCGCGGTAA, R: GGACTACHVGGGTWTCTAAT (Kozich et al., 2013), an 18S rDNA amplicon that targeted single‐celled eukaryotes, F: TCTCAGGCTCCYTCTCCGG, R: AAGCCATGCATGYCTAAGTATMA (Dollive et al., 2013), and a region of the 16S rDNA gene designed to bind preferentially arthropod DNA, F: TGAACTCAGATCATGTAA, R: TTAGGGATAACAGCGTAA (Kartzinel & Pringle, 2015). We pooled the resulting PCR products for each individual host. We prepared the amplicons for sequencing using the NEBNext Ultra 2 DNA prep kit for Illumina, with custom barcodes following those suggested by Peterson, Weber, Kay, Fisher, and Hoekstra (2012). The libraries were sequenced on a MiSeq platform at the University of Michigan Microbiome Core Facility.

### 2.3 Sequence curation pipeline

We used the program mothur v.1.39.5 to prepare an error‐checked and taxonomically identified set of reference 16S rDNA bacterial sequences for later individual‐specific analysis (Schloss et al., 2009). We made contigs from our paired‐end sequences, selected appropriately sized fragments, and removed low‐quality sequences and those with homopolymers over eight basepairs long. We then aligned the sequences against the reference bacteria in the Silva v. 128 database (Pruesse, Peplies, & Glöckner, 2012; Quast et al., 2012; Yilmaz et al., 2014), and removed the sequences that did not align well. We used vsearch v2.3.4 (Rognes, Flouri, Nichols, Quince, & Mahé, 2016) to check sequences for chimeras and clustered all sequences in the database to 97% similarity, retaining one sequence per cluster in the final database. Each sequence in our curated database is identified by the taxonomic identity of the Silva reference sequence with which it is most closely aligned.

We used vsearch to make contigs for the sequences from each host and then aligned the contigs to our bacterial database. We used a custom script written for R 3.5.1 to parse the resulting alignment files into a host‐by‐OTU community matrix. We retained the results from all hosts with more than 10,000 total sequences in the community matrix, and rarefied the matrix to 10,000 sequences per host.

To identify microbial eukaryote and arthropod prey sequences, we made contigs using vsearch, and then selected sequences in the correct size range for the target amplicon using R. We dereplicated the sequences and checked for chimeric sequences using vsearch. To identify sequences to taxon, we use the discontinuous megablast algorithm to search against the NCBI database. For both amplicons, we removed putative sequences that were less than 90% similar to any reference sequence in the NCBI Blast database, or those that aligned to more than one order. We then realigned our contigs to the curated eukaryote and arthropod databases, by following the same procedure we used for the bacterial amplicons. For each amplicon, we made a host‐by‐eukaryote order community matrix, populated by the number of sequences amplified for that order from each host. We repeated the procedure with the arthropod prey sequences.

### 2.4 Bacterial community structure

To identify the taxonomic structure of the microbiome, we found the proportion of bacterial OTUs made up by each of the four most common bacterial phyla in each host. To assess the impact of host species, arthropod prey, and eukaryotic microbes on bacterial community composition, we performed an NMDS in the R package “vegan 2.5‐4” (Oksanen et al., 2018). The function calculated Bray‐Curtis distances in bacterial microbiome between all pairs of hosts, and then projected those distances onto two dimensions. We used the function “envfit” to fit binary presence‐absence vectors of host species, eukaryote orders, and diet items onto the NMDS space. We calculated the significance of the vectors using randomization. For the eukaryotes and diet items that predicted significant differentiation at a *p*‐value of .05, we calculated the directionality and strength of the predictor relative to the NMDS axes. We performed 68 “envfit” comparisons. Our Bonferroni‐corrected significance level was therefore .0007. None of our randomization‐based significance levels reached .0007. To capture nonsignificant but potentially ecologically relevant trends, we report vectors that reached a significance level lower than .05, as well as vectors for the two host species.

### 2.5 Bacterial community structure

To identify the ecological interactions between bacterial OTUs, eukaryotes, host species, and diet items, we used the R package “cooccur” (Griffith, Veech, & Marsh, 2016). We used each of the 27 hosts as a site in which two taxa might both occur. For each pair of taxa, we took the observed number of times each occurred in the dataset and the observed number of times they overlapped in the same host. We used the “cooccur” function to calculate the probability of that pair occurring together fewer or more times than observed. We used taxa that occurred in two or more hosts for this analysis.

We visualized cooccurrence relationships by making undirected graphs in the R package “igraph” (Csardi & Nepusz, 2006). We plotted undirected graphs with bacterial families, eukaryotes, and diet items as nodes and edges representing pairwise taxon interactions that met a .01 significance level. We chose .01 as a cutoff significance level due to the discrete nature of possible significance values in the “cooccur” algorithm. With only 27 host animals, we had limited ability to detect highly significant values. With our large number of comparisons, a Bonferroni‐corrected significance cutoff of .05 would be 6.3 × 10^−8^. While we find *p*‐values of zero in our dataset, the smallest non‐zero detectable *p*‐value was 1 × 10^−5^. Given this limitation, our chosen cutoff value allowed us to examine potentially ecologically relevant pairwise interactions while respecting the limitations inherent in applying this algorithm to our dataset. To better understand how the observed patterns of overlap in our dataset differed from expected patterns in a truly random dataset, we randomized the hosts in which each bacterial OTU occurred and recalculated the cooccur function for the randomized dataset five times.

### 2.6 Bacterial function and ecological interactions

We found a large number of higher‐than‐expected overlaps between OTUs within bacterial families. We hypothesized that unaccounted‐for variation within hosts was leading similar OTUs to cluster together. To better understand the clustering, we used bacterial function as a proxy for ecological similarity between OTUs. To assign functional profiles to bacterial OTUs, we used the KEGG Orthology database, a database of genomic sequences mapped to biological functions, including bacterial metabolic functions (Kanehisa et al., 2016). The KEGG database assigned metabolic pathways to 16S rDNA genetic sequences from reference bacteria in which those pathways are known to occur. Under the assumption that phylogenetically similar bacteria will share metabolic capabilities, we extrapolated from these reference sequences to assign a metabolic profile to the bacteria in our dataset. The KEGG database matched functional profiles with 16S rDNA barcodes from the GreenGenes database. We realigned our curated 16S rDNA barcode library with the GreenGenes database using vsearch. We converted our function by OTU matrix into a presence–absence matrix, which we used for all further analyses.

Since efforts in laboratory investigation of bacterial functional pathways have not been taxonomically balanced, these data were susceptible to false negatives, as functional pathways could occur in a given taxon in which they have not yet been identified. Due to this concern, we calculated the pairwise Bray‐Curtis distance between the presence–absence functional profiles of each pair of OTUs. We chose the Bray‐Curtis metric because it is robust to missing data (Bray & Curtis, 1957; Faith, Minchin, & Belbin, 1987). We regressed the inverse of the functional distances (a metric of functional similarity) against an observed versus expected cooccurrence metric. We first calculated the percent difference between the observed and expected numbers of cooccurrences between every pair of bacterial OTUs generated from our “cooccur” analysis. High positive numbers indicated higher‐than‐expected cooccurrence, while high negative numbers indicated lower‐than‐expected cooccurrence.

We identified the eukaryotes, host species, and diet items that showed either a greater‐than‐expected or less‐than‐expected cooccurrence pattern with a probability value of .01 or less with two or more bacterial OTUs. To provide a more granular understanding of the metabolic functions of the interacting OTUs, we broke our functional profile matrix into 12 subsets. We sorted the KEGG orthologs into functional groups using a hierarchical system of increasingly more specific descriptors of the function of a pathway. We chose the second most general level of descriptors, and used only those sets of descriptors that had eight or more unique functions within them in our dataset.

We found pairwise Bray‐Curtis distances between every bacterial OTU in each functional subset. For each set of OTUs that significantly cooccurred with a host, eukaryote, or diet item at a value of .05 or less, we calculated the mean nearest neighbor distance in this functional space. We used this as a measure of clustering by the OTUs within function space. Using the R package “usedist,” we calculated the displacement between the centroid of the interacting OTUs and the centroid of all OTUs (Bittinger, 2017). The displacement is a metric of the differentiation between the functional profile of the interacting OTUs and the “average” functional profile of the entire microbiome. To determine whether the observed mean nearest neighbor and displacement values were significantly different from a null expectation, we randomly drew subsets containing the same number of OTUs as each of our significantly interacting subsets. For each functional subset by eukaryote or diet item comparison, we drew 5,000 random subsets of OTUs, and recalculated the nearest neighbor and displacement statistics. We found the percentage of random subsets that were more clustered and more displaced than our interacting OTU groups.

## 3 RESULTS

### 3.1 Bacteria, eukaryotes, and diet items recovered

**Figure 1:**
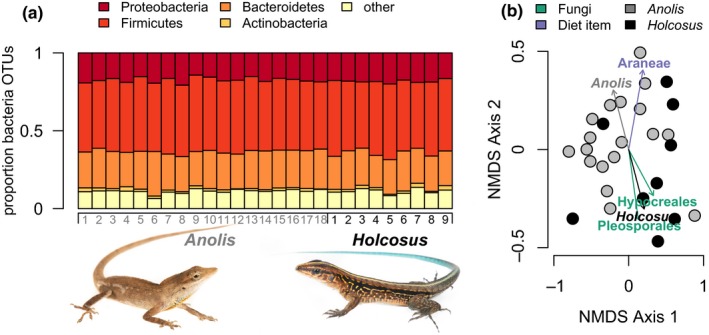
Gut microbiomes of 18 silky anole, *Anolis sericius,* and 9 rainbow ameiva, *Holcosus undulatus,* lizard hosts show similar proportions of bacterial OTUs in each of the four widespread bacterial phyla (a). One food item and two eukaryotic microbes show significant fit to an NMDS based on pairwise Bray‐Curtis dissimilarity between the bacterial communities of each host (b). Photo credit for lizard images to Jose Martinez Fonseca

The most common bacterial phyla in our samples were Proteobacteria, Bacteroidetes, Actinobacteria, and Firmicutes (Figure 1a). They made up consistent proportions of the OTUs in the microbiome across hosts. Rare phyla included Acidobacteria, Chlamydiae, Fusobacteria, Gemmatimonadetes, Lentisphaerae, Planctomycetes, Spirochaetes, and Verrucomicrobia.

We amplified 41 eukaryotic microbial orders from our scats. Fungi were the most common eukaryotic microbe across our lizard hosts (Table 1). Two fungi, Basidiobolales (from the fungal higher taxon Zoopagomycota) and Mucorales (from the higher taxon Zygomycota), occurred in nearly every host. Other common taxa included Eugregarinoria, an apicomplexan parasite, and Capnodiales and Eurotiales, both in the fungal taxon Ascomycota. The protists Tritrichomonadida and Cercomonadida, the Apicomplexans Neogregarinorida and Eucoccidiorida, and the nematode Rhabditidia were present in multiple hosts but were less widespread. The dominance of fungi may be related to the design of the eukaryotic 18S rDNA primer we used (Dollive et al., 2013). We suggest investigating a range of primers or using primer‐free approaches in the future to quantify the eukaryotic microbiome.

**Table 1:** Number of sequences per host in the four most abundant bacterial phyla, and in select eukaryote and diet item orders, and the number nontarget sequences amplified by the eukaryote and diet item primers in each host

Host	Proteobacteria	Firmicutes	Bacteroidetes	Actinobacteria	Basidiobolales	Mucorales	Tritrichomonadida	Eugregarinorida	Orthoptera	Diptera	Hemiptera	Coleoptera	Hymenoptera	Psocoptera	Araneae	Non‐target
Anolis 1	12,732	10,934	2,777	164	259	57	31	3	13	6	1	1	1	0	0	2,112
Anolis 2	1,592	10,507	13,389	168	633	2	354	5	1	0	0	0	0	0	0	3,223
Anolis 3	7,645	9,962	14,143	70	276	2	0	0	18	2	0	0	0	475	0	553
Anolis 4	11,550	10,752	2,966	621	2,098	6	0	7	0	1,108	0	0	15	0	0	713
Anolis 5	7,086	7,713	9,320	18	1,446	479	10	0	0	0	0	0	450	0	0	209
Anolis 6	10,190	2,970	15	594	0	577	0	0	0	1	0	0	0	0	1	603
Anolis 7	3,736	3,714	5,045	121	993	176	236	38	2	1	2	0	0	3	0	38
Anolis 8	4,332	2,607	6,134	4	723	8	0	1	0	0	0	0	0	0	6	486
Anolis 9	11,905	3,826	9,802	165	84	9	0	1	0	0	46	0	0	0	0	1,639
Anolis 10	11,523	2,961	12,882	430	8	0	0	16	3	0	0	13	1	0	1	1,525
Anolis 11	2,812	5,471	6,578	116	54	5	1	5	0	0	0	0	14	0	0	1,215
Anolis 12	105,187	56,219	6,954	141	331	2,100	3	0	9	0	0	0	0	0	0	16,069
Anolis 13	7,459	3,175	7,338	4	768	1,218	0	0	0	2,860	0	0	0	0	0	119
Anolis 14	7,296	12,421	14,393	402	1,454	65	1,582	0	0	0	0	0	4	0	0	4,248
Anolis 15	6,244	9,269	10,347	68	153	1	0	4	6	1	0	1	1	0	0	2,136
Anolis 16	6,195	4,686	7,541	137	247	886	78	0	1	2	0	0	0	0	0	345
Anolis 17	2,871	3,530	6,324	113	709	1,291	330	0	0	0	0	0	3	0	0	190
Anolis 18	22,923	2,814	2,543	36	4	1	0	7	1	1	0	1	0	0	55	2,006
Holcosus 1	7,960	1,571	11,258	68	777	0	0	5	0	0	0	1,661	0	0	0	1,201
Holcosus 2	31,963	3,398	9,788	80	853	417	0	5	1	0	1	2,747	19	0	1	2,031
Holcosus 3	12,542	10,786	3,849	47	3	24	0	1	2	0	0	0	0	2,313	0	1,517
Holcosus 4	3,021	10,128	14,511	46	0	0	0	2	0	0	4	0	0	0	0	393
Holcosus 5	7,433	3,637	4,077	8	1,016	249	0	0	11	1	0	0	0	0	1	245
Holcosus 6	5,994	4,999	5,202	26	3	1,288	0	2	1	0	0	2	1	0	0	1,469
Holcosus 7	9,777	14,347	550	32	318	5	0	1	1	0	0	0	0	0	0	1,715
Holcosus 8	33,691	4,018	733	226	225	6	2	10	8	0	0	0	6	0	0	4,088
Holcosus 9	6,200	9,018	86	82	1	11	2	0	3,024	0	0	0	0	0	21	3,751

We amplified a total of 14 arthropod orders from our lizard scats. The most widespread were Diptera (flies), Psocoptera (booklice), Orthoptera (crickets), Lepidoptera (butterflies and moths), Hymenoptera (wasps, bees, and ants), Hemiptera (true bugs), and Coleoptera (beetles). We also amplified mites (Mesostigmata) and spiders (Araneae) (Table 1, Table S1).

### 3.2 Bacterial community structure

We calculated pairwise Bray‐Curtis dissimilarity between the bacterial microbiome communities in each host animal and visualized the matrix on two NMDS axes (Figure 1b). The A*nolis* host species vectors was below our .05 reporting cutoff (*p* = .049), while the *Holcosus* host species vector approached our cutoff (*p* = .059). Bacterial communities from hosts that contained the fungus Hypocreales trended differently from those that did not (*p* = .030), as did those that had the fungus Pleosporales compared to those that did not (*p* = .041; Figure 1b). Hosts that had eaten spiders (*p* = .013) also trended differently from those that had not.

Our “cooccur” analysis identified 842,101 possible pairs of taxa in our dataset, of which 792,432 were bacteria–bacteria pairs. A total of 12,947 bacteria–bacteria pairs occurred in the same host more frequently than expected at a significance value of .01, and 703 pairs occurred in the same host less frequently than expected at the same significance level. To better understand how patterns of pairwise overlap in our dataset differ from random expectations, we compared the cooccurrence *p*‐values calculated for our bacterial OTUs to an expected distribution of *p*‐values for a truly random dataset. We found that under strictly random interactions, we would expect between 1,364 and 1,473 pairs of bacteria to overlap more than expected at a significance value of .01, and between 384 and 457 pairs to overlap less frequently than expected at the same significance value. These values, particularly for greater‐than‐expected overlap, are considerably smaller than the empirical values we observed in our dataset.

**Figure 2:**
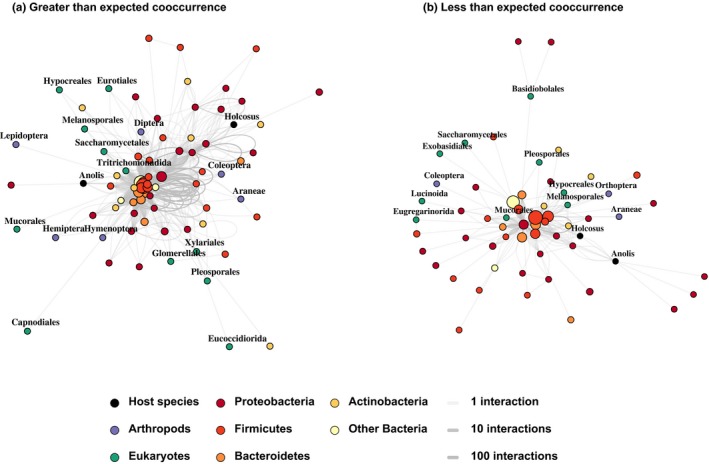
Graphs of greater‐than‐expected and lower‐than‐expected levels of overlap (significance value cutoff of .01) between pairs of taxa show contrasting patterns. Greater‐than‐expected overlap occurs frequently in our dataset, and greater‐than‐expected overlap between OTUs of a single bacterial family is common (a). Lower‐than‐expected levels of overlap is rarer, and lower‐than‐expected overlap within bacterial families is rare (b)

Our “igraph” visualization of the “cooccur” results show that many positive interactions center around OTUs in the Bacteroidetes and Firmicutes phyla (Figure 2a). Members of the same bacterial family frequently occur in the same host more often than would be expected by chance (loops out and back to nodes in Figure 2a). More abundant families tend to be involved in more positive interactions. Pairs of taxa cooccurring less frequently than expected showed a contrasting pattern. Bacterial family abundance is not a good predictor of the number of negative interactions, with one relatively small Proteobacterial family showing the most connectivity (Figure 2b). Only one bacterial family has negative cooccurrence between two of its member OTUs.

### 3.3 Bacterial function and ecological interactions

**Figure 3:**
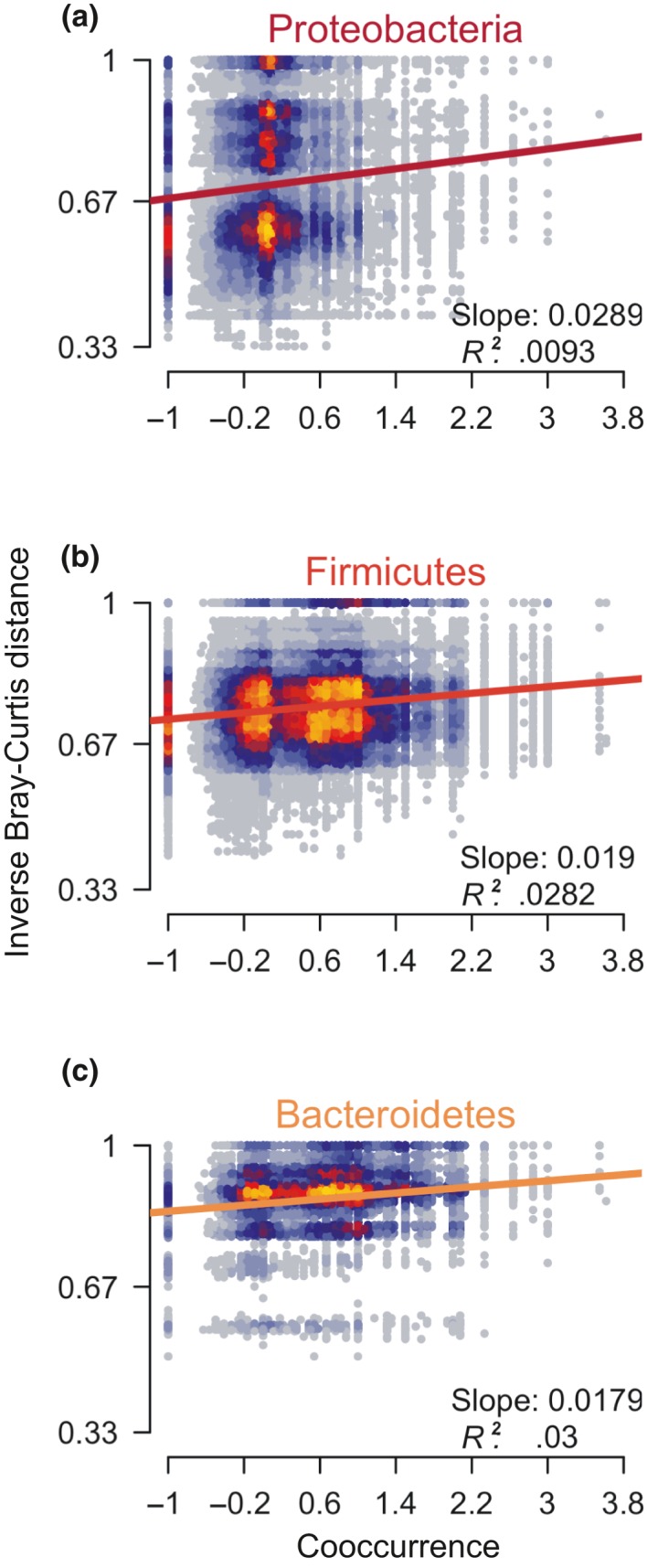
Bacterial OTUs that cooccur more frequently are more likely to have similar functional profiles. The *X*‐axis shows the percentage difference between the observed and expected cooccurrence values for each pair of OTUs within the three most abundant bacterial families, Proteobacteria (a), Firmicutes (b), and Bacteroidetes (c). The *Y*‐axis shows the inverse value of the Bray‐Curtis dissimilarity in functional profile between each pair of OTUs

Within each major bacterial phylum, we found greater‐than‐expected cooccurrence between pairs of bacteria was significantly positively correlated with functional similarity (Figure 3). Between phyla, no such relationship existed (Table S2).

**Figure 4:**
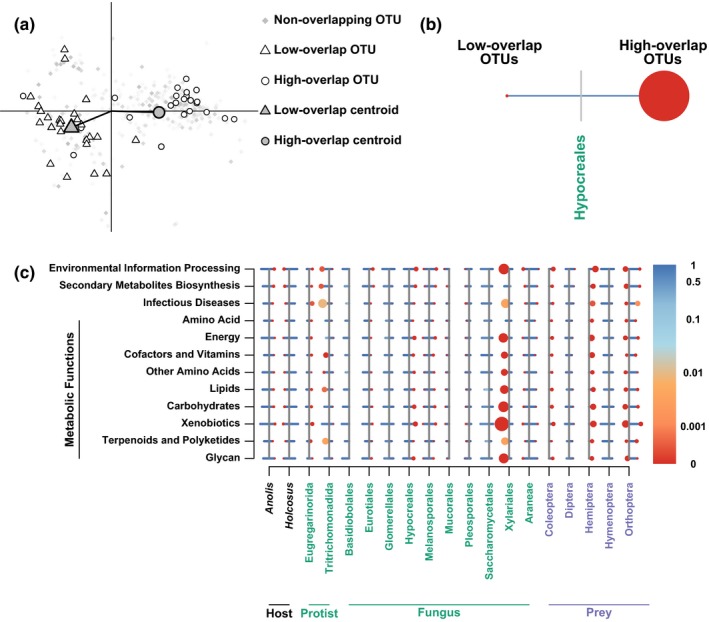
Bacterial OTUs that significantly overlap with eukaryotes or diet items cluster in function space. (a) For each bacterial functional category, we create a function space from pairwise distances between bacterial functional profiles. Within that space, we find the locations of bacterial OTUs that overlap more or less than expected with a given eukaryote. To summarize these data, we find the mean nearest neighbor distance between each group, and find the displacement of the centroid of each group from the centroid of the entire function space. (b) To represent clustering and displacement of, we use two points. The distance of the points from a line represents the displacement from the main centroid, and point size represents mean nearest neighbor distance. The color of the points and lines, from a log‐scale color ramp, represents the proportion of the 5,000 randomizations that had smaller mean nearest neighbor distance or smaller displacement than the real data. (c) Across all comparisons, functional profiles of interacting OTUs are more highly clustered than would be expected at random

We considered the functional profiles of bacterial OTUs that occurred more or less frequently than expected with eukaryotic microbes and diet items. We divided the KEGG orthologs into subsets of broadly similar functions. Nine subsets involved metabolism of some type of energy source, and the remaining three were related to infectious diseases, biosynthesis of secondary metabolites, and environmental information processing. Eleven eukaryotes (nine fungi and two protists) had two or more OTUs that cooccurred at a significance value of .01. Six diet item categories (flies, spiders, true bugs, wasps, beetle, and crickets) had two or more significantly cooccurring OTUs. Both host species had OTUs that cooccurred at a significance value below .01. We excluded all eukaryotes and diet items with only one interacting bacterial OTU, because we could not calculate functional clustering in these cases. We found that our focal OTU groups were always more clustered than 98% of random draws (Figure 4c). Displacement of the focal OTU centroid was much more variable, both within and between functional subsets and eukaryotes or diet items.

## 4 DISCUSSION

Our study is the first profile of lizard gut microbiomes to incorporate eukaryotic community members and food items being digested at the time of sampling. By assessing all three factors in tandem, we are able to test hypotheses about the ecological processes that underly the bacterial microbiome's functional shifts in reaction to food items and eukaryotes in wild hosts. We find that functionally and phylogenetically similar bacteria tend to cluster in host animals. We hypothesize that subsets of the bacterial microbiome respond to the diet items being digested by the host at the time of sampling and to the eukaryotic microbes present in the host. Bacteria that cooccur with diet items or eukaryotes to a greater or lesser extent than expected tend to be highly clustered in function space.

### 4.1 Microbiome taxonomic structure

Proteobacteria, Firmicutes, and Bacteroidetes are the most abundant taxa, with the relative number of OTUs from each being consistent from host to host, even across host species. These results are similar to previous work that also found squamate reptile gut microbiome communities dominated by Proteobacteria OTUs, with a consistent presence of Firmicutes, Bacteroidetes, and Actinobacteria (Colston & Jackson, 2016; Colston et al., 2015; Kohl et al., 2017). We note that we did not perform a complete assessment of the gut microbiome community, as we focus only on taxa that can be amplified from scats. The intestinal mucosal layer is the interface between a host and their microbiome, and contains a distinct microbial community compared to that found in the intestinal lumen (Jacobs & Braun, 2014). We focus on scat microbiome to best capture microbe–food item interactions. However, future work should include examination of different microhabitats within the squamate gut. We found that neither the food items lizards are digesting at the point of measurement nor the eukaryotes in the gut community significantly correlate to the bacterial community structure. Further study is necessary to understand how the eukaryotic microbiome interacts with the bacterial microbiome and the health of the host.

The eukaryotic microbiome of the lizards in our study is dominated by fungi. The two most widespread orders were in the taxon Zoomycota. Zoomycota are frequently associated with insects, so the lizards may have consumed them along with their insect prey (Vega & Blackwell, 2005). Mammals generally have eukaryotic microbiomes dominated by either Ascomycota or Basidiomycota, but the two taxa do not cooccur (Hoffmann et al., 2013). In lizards, both Zoomycota and Ascomycota frequently occur in single hosts, with Basidiomycete fungi also cooccurring with both in four hosts. Ascomycetes had the largest diversity in our samples at the order level, with eleven separate orders recorded.

### 4.2 Bacterial microbiome ecological interactions

The majority of OTUs in the bacterial microbiome showed cooccurrence patterns consistent with neutral community assembly. Most of the exceptions were closely related, functionally similar OTUs that occurred in the same host more often than expected (Figures 3 and 4). This pattern is consistent with hosts recruiting a suite of functionally similar OTUs to help them accomplish a specific metabolic task. It is also consistent with ecologically similar OTUs, all finding success when they encounter an environment (host) that is favorable to them. When a bacterial OTU often cooccurs with a specific food item, it may have been introduced to the microbiome when the host consumed that item. Negative cooccurrence patterns (Figures 2b and 4c) cannot be explained through such a mechanism. More study is necessary to determine the physiological and ecological processes that underly negative cooccurrence in the microbiome.

### 4.3 Bacterial microbiome functional characteristics

We propose an analogy from the small‐scale shifts in functional profile shown by the microbiome to phenotypic plasticity, in which external conditions trigger an organism to express one of a set of possible phenotypes. However, plastic phenotypes require a specific genetic underpinning to originate and be maintained in a lineage. This process has been well‐studied in a few model taxa (Levis & Pfennig, 2016). We are particularly interested in making an analogy from the process of channelization of phenotypic plasticity to the processes that lead to divergence in gut microbiome communities between different host species or ecotypes. Phenotypic channelization can occur when members of a population are more successful in one of the possible phenotypic states relative to the other. The more successful state can then become channelized, fixing that morph in the species and losing the plastic response to the initial trigger signal (von Heckel, Stephan, & Hutter, 2016). Extrapolating from the process of channelization may prove useful in developing a mental model of gut microbiome evolution at broader phylogenetic scales, although the analogy is imperfect.

Animal microbiome composition is often best predicted by the diet of the host, when a wide variety of hosts with distinctly different diet niches are considered. Mammals are prime examples: the taxonomic composition of herbivore microbiomes is markedly different from that of carnivores (Ley et al., 2008). When considering groups of animals with more similar dietary niches, the precise composition of the diet becomes less predictive of the composition of the microbiome (Baxter et al., 2015; Phillips et al., 2017). Diet composition remains predictive of the functional profile of the gut microbiome at much finer degrees of diet resolution (Phillips et al., 2017).

We hypothesize that, as a lineage shifts from one dietary niche into a substantially different niche, the shift first occurs at the level of the functional profile of the microbiome (Phillips et al., 2017). If a subset of bacterial taxa, such as those observed cooccuring with specific diet items in this study, is more efficient at providing the functions necessary to the new diet niche, they may become “channelized” in the host lineage, leading to a consistent and identifiable shift in composition at the taxonomic level of the microbiome. Identifying the precise level of diet resolution at which the “channelization” of the microbiome community takes place will require extensive investigation of natural host communities. One potential avenue would be to identify when a microbe shifts between the “core” and “peripheral” microbiome in a lineage (Shapira, 2016). Microbes could change in importance to their host over time, while staying present in the microbiome.

Understanding the genetic basis of “channelization” in the host and the microbiome is a further area of inquiry that will require extensive future work. One major factor that may delay the microbiome taxonomic turnover associated with “channelization” into new diet niches is that the microbiome provides a number of services to the host beyond those involved in digestion, including disease resistance and parasite management. The trade‐offs inherent in microbiome function are relatively unique to this community, and may not have a direct analogy in other systems. An abrupt evolutionary change by a microbiome to adapt to a novel food source might damage the effectiveness of other services, thereby reducing the overall fitness of the host. Gut microbiome shifts in taxonomy require trade‐offs between the many functions the microbiome performs, so shifts in function to accommodate food sources in real time may be a critical component of the successful association between gut microbiome and host.

## CONFLICT OF INTEREST

None declared.

## AUTHOR CONTRIBUTIONS

Iris Holmes directed sequencing, analysis, and manuscript writing. Ivan Monagan collected the samples, and participated in sequencing, analysis, and writing. Daniel Rabosky participated in analysis and writing. Alison Davis Rabosky participated in analysis and writing.

We appreciate the support of the University of Michigan Museum of Zoology in funding open access payments for this publication.

## Data Availability

All fastq files are available from NCBI's Sequence Read Archive, from BioProject PRJNA564442. Tables S1 and S2 are available in csv format from the DataDryad repository (https://doi.org/10.5061/dryad.r1h8gm4).
