# Supplementary material for: Metabolically similar cohorts of bacteria exhibit strong cooccurrence patterns with diet items and eukaryotic microbes in lizard guts
Source: Ecol Evol. 2019 Oct 23;9(22):12471–81. doi: 10.1002/ece3.5691 (PMC6875663; doi:10.1002/ece3.5691)
Supplement: Supplementary file 2 [file ECE3-9-12471-s002.pdf]

Table_S2

	slope	R.2
Proteobacteria_Proteobacteria	0.02890788849657	0.009343785990825
Proteobacteria_Firmicutes	0.005268602403898	0.003379468169962
Proteobacteria_Bacteroidetes	0.005903358135081	0.005442298439523
Firmicutes_Proteobacteria	0.005268602403898	0.003379468169962
Firmicutes_Firmicutes	0.018997550015625	0.028176481049513
Firmicutes_Bacteroidetes	0.002125258856078	0.002397785484486
Bacteroidetes_Proteobacteria	0.005903358135085	0.005442298439523
Bacteroidetes_Firmicutes	0.002125258856079	0.002397785484486
Bacteroidetes_Bacteroidetes	0.017902184899718	0.029994335510288
